# Supplementary material for: The effects of aging and hearing impairment on listening in noise
Source: iScience. 2024 Feb 20;27(4):109295. doi: 10.1016/j.isci.2024.109295 (PMC10981015; doi:10.1016/j.isci.2024.109295)
Supplement: Table S1. The subset of items from the HHIE administered in study, related to the subjective hearing impairment questionnaire section of method details in STAR Methods [file mmc1.pdf]

iScience, Volume *27*

Supplemental information

The effects of aging and hearing impairment on listening in noise

Ádám Boncz, Orsolya Szalárdy, Péter Kristóf Velősy, Luca Béres, Robert Baumgartner, István Winkler, and Brigitta Tóth

Supplemental Information

Table S1. The subset of items from the HHIE administered in study, Related to the Subjective hearing impairment questionnaire section of Method Details in STAR Methods.

1. Does a hearing problem cause you to feel embarrassed when you meet new people?
2. Does a hearing problem cause you to feel frustrated when talking to family members?
3. Do you have difficulty hearing when someone speaks in a whisper?
4. Do you feel handicapped by a hearing problem?
5. Does a hearing problem cause you difficulty when visiting friends, relatives, or neighbors?
6. Does a hearing problem cause you to attend religious services less often than you would like?
7. Does a hearing problem cause you to have arguments with family members?
8. Does a hearing problem cause you difficulty listening to television or radio?
9. Do you feel that difficulty with your hearing limits/hampers your personal or social life?
10. Does a hearing problem cause you difficulties when in a restaurant?
